# Novel Approaches to Understanding Education and Later-Life Cognitive Health

**DOI:** 10.1093/geroni/igaf122.1511

**Published:** 2025-12-31

**Authors:** Lindsay Kobayashi, Jennifer Manly

## Abstract

Education is the strongest known modifiable factor that reduces dementia risk. However, there remain several unanswered questions about this relationship, which limit the design of policies to optimize the role of educational systems in promoting the cognitive health of populations. This symposium presents a suite of epidemiological studies using complementary designs and analytical approaches to uncover new insights into the complex role of education in later-life cognitive health across diverse older populations. First, Emma Nichols will present a series of analyses designed to probe the counterintuitive finding of faster cognitive decline amongst older adults with higher educational attainment in a population-based cohort study of aging in India. Results demonstrate how regression to the mean may bias longitudinal studies of education and cognitive decline. Second, Mateo Farina will present on the interactive roles of relative and absolute educational attainment in shaping later-life cognitive function among men and women in five high- and middle-income countries, using an intersectionality framework. Third, Vicky Chen will examine the role of cerebrovascular and neurodegenerative mediators linking educational attainment with cognitive decline among men and women in a comparative study of older adults in Korea and the United States. Fourth, Sirena Gutierrez will investigate how literacy, numeracy, and educational attainment contribute to cognitive aging disparities between Indigenous and non-Indigenous older adults in Mexico. Jennifer Manly will serve as the discussant, integrating findings to discuss the complex role of education and later-life cognitive health across these globally diverse aging populations.

